# Functional dyspepsia overlapping with other functional gastrointestinal disorders: a bibliometric and visualization analysis

**DOI:** 10.3389/fmed.2026.1742579

**Published:** 2026-04-08

**Authors:** Mingming Fan, Dexin Wang, Kunpeng Yu, Xiaojun Cai, Jiankun Cui, Fei Li, Cong Sun, Aofei Wang, Yong Li, Qingyan Liu

**Affiliations:** 1 Nangang Branch of Heilongjiang Academy of Traditional Chinese Medicine, Harbin, China; 2 Cell Biology Laboratory, Key Laboratory of Liver and Kidney Disease of the Ministry of Education, Clinical Key Laboratory of TCM of Shanghai, Institute of Liver Diseases, Shuguang Hospital Affiliated to Shanghai University of Traditional Chinese Medicine (TCM), Shanghai University of TCM, Shanghai, China; 3 Shenyang Anorectal Hospital, Shenyang, China; 4 Xiangan Branch of Heilongjiang Academy of Traditional Chinese Medicine, Harbin, China; 5 Heilongjiang University of Traditional Chinese Medicine, Harbin, China; 6 Second Affiliated Hospital of Heilongjiang University of Chinese Medicine, Harbin, China

**Keywords:** bibliometric analysis, functional dyspepsia, functional gastrointestinal disorders, overlap, visualization analysis, disorders of gut-brain interaction

## Abstract

**Background:**

Functional dyspepsia (FD) overlapping with other functional gastrointestinal disorders (FGIDs) has been a prominent topic in gastroenterology. Knowledge in this area has evolved rapidly over the past two decades. Using bibliometric approaches, this study aimed to evaluate the research landscape on FD overlapping other FGIDs over the last 20 years and to identify major themes and emerging topics.

**Objectives:**

To apply network-based bibliometric methods to comprehensive summarize research progress and trends on FD overlapping other FGIDs, thereby providing evidence and guidance for further studies.

**Methods:**

On October 1, 2025, we searched the Web of Science Core Collection (WoSCC) for publications from 2005 to 2025 related to FD overlapping other FGIDs. Records were imported into VOSviewer, CiteSpace, and the bibliometrix R package to extract metadata and conduct bibliometric analyses, including annual output, countries/regions, authors, institutions, journals, citation counts, and keywords. To ensure robustness and generalizability, equivalent searches were conducted in Scopus and PubMed using the same keyword set, time span, and eligibility criteria. Cross-database validation assessed concordance in temporal trends, thematic foci, and country rankings.

**Results:**

A total of 3,030 WoSCC records were retrieved. Nicholas J. Talley ranked first by number of publications (*n* = 188), followed by Jan Tack (*n* = 95). The top three institutions were Mayo Clinic, USA (*n* = 170), KU Leuven, Belgium (*n* = 123), and the University of Newcastle, Australia (*n* = 120). The USA contributed the largest number of publications (*n* = 820). The three most productive journals were *Neurogastroenterology and Motility* (*n* = 209), *Alimentary Pharmacology & Therapeutics* (*n* = 106), and *American Journal of Gastroenterology* (*n* = 96). Importantly, multi-database validation demonstrated high consistency in annual publication trends, substantial overlap among high-frequency keywords, and stable geographic and disease-focused research emphases.

**Conclusion:**

This study comprehensive maps the evolution of research on FD overlapping other FGIDs over the past two decades, providing researchers with an updated overview and fresh insights. Our findings facilitate a comprehensive review of the field and offer a reference to inform future investigations.

## 1 Introduction

Functional dyspepsia (FD) is one of the most prevalent subtypes of functional gastrointestinal disorders (FGIDs; currently termed disorders of gut-brain interaction, DGBIs). According to the Rome IV criteria, FD can be further classified into epigastric pain syndrome (EPS) and postprandial distress syndrome (PDS) (1). In real-world practice, multiple FGIDs commonly overlap; FD frequently coexists with gastroesophageal reflux disease (GERD), irritable bowel syndrome (IBS), and functional constipation (FC) (2). Although GERD is structurally distinct from DGBIs, the 2022 APAGE Clinical Practice Guidelines explicitly identify “FD overlapping with GERD” as a critical clinical phenotype requiring specific management strategies. Such overlap substantially affects therapeutic response and patient outcomes. However, many clinicians tend to focus on the most prominent symptom or address each symptom in isolation, which may overlook shared mechanisms across different FGIDs (3). As a major component of digestive diseases, FGIDs are highly prevalent and impose a significant burden on quality of life, yet their etiologies and pathophysiology remain incompletely understood and continue to pose major challenges (4).

Bibliometrics, an important quantitative tool, has been widely applied across disciplines (5). By employing mathematical and statistical methods to assess the volume, distribution, structure, and temporal evolution of scholarly output, bibliometric analysis characterizes the development of a field, publication trends, key contributors, and national/institutional productivity. It also helps identify future hotspots, emerging fronts, and the underlying knowledge structure, thereby providing researchers and clinicians with a visual, field-specific overview (6). In recent years, with the maturation of specialized tools such as CiteSpace, VOSviewer, and the bibliometrix R package, bibliometrics can more efficiently process large bodies of literature, delineate disciplinary trajectories, evaluate scholarly influence, and forecast frontiers (7). Against this background, we conducted a bibliometric analysis of research on FD overlapping other FGIDs over the past two decades, aiming to help clinicians better grasp advances in the field and to inform future investigations (8).

## 2 Methods

### 2.1 Data sources

We comprehensive searched the Web of Science Core Collection (WoSCC) for studies on FD overlapping with other FGIDs. All searches and data extraction were completed on the same day (October 1, 2025) to minimize potential bias from database updates. The time span was restricted to January 1, 2005 through September 30, 2025. Eligible records were limited to Articles and Reviews published in English. The WoSCC topic search combined controlled terms and free-text synonyms as follows (5–8) (adjusted as needed to conform to each database’s syntax): TS = ((“Dyspepsia*” OR “Indigestion*” OR “functional dyspepsia” OR FD OR “non-ulcer dyspepsia” OR “idiopathic dyspepsia” OR “non-organic dyspepsia” OR “non-ulcerative dyspepsia”) AND (“gastroesophageal reflux disease” OR GERD OR “irritable bowel syndrome” OR IBS OR “functional constipation” OR FC)) AND PY = (2005–2025) AND DT = (Article OR Review) AND LA = (English). Consistent with established bibliometric guidelines (9), we adopted a broad search strategy without explicitly including terms such as ‘overlap’ or ‘comorbidity’ in the initial query. This approach was chosen to minimize Type II errors (missing relevant studies) and ensure a comprehensive dataset, accepting the trade-off of requiring rigorous post-hoc refinement to filter for specificity.

### 2.2 Search strategy

**Figure 1:**
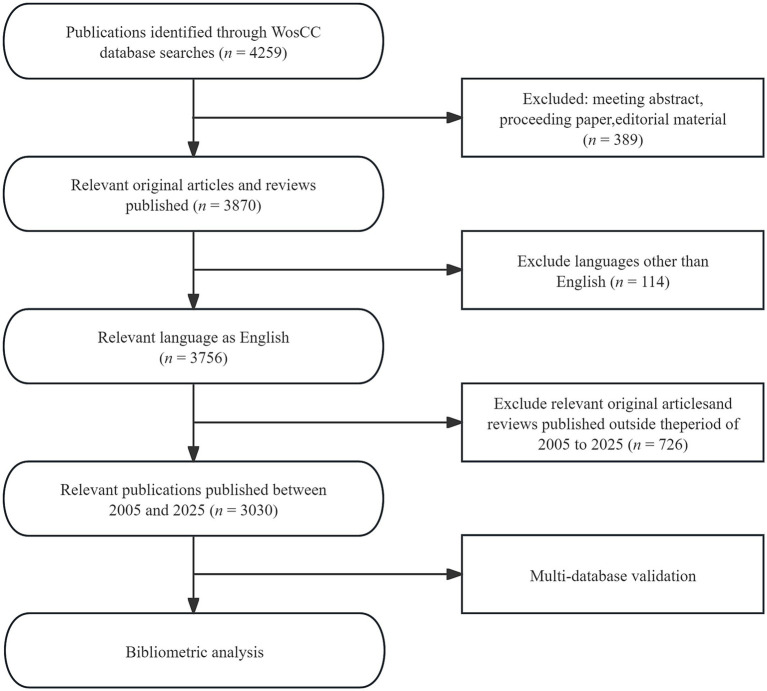
Flowchart of inclusion and exclusion of publications on functional dyspepsia overlapping other functional gastrointestinal disorders.

After the initial retrieval, we excluded duplicates, early online/early access items, records with incomplete metadata, conference abstracts, and studies not relevant to the topic. A total of 3,030 records were finally included; the screening process is illustrated in Figure 1. Given the search cutoff of October 1, 2025, studies formally published within 2025 up to that date were included to reflect the most up-to-date progress. For database selection, WoSCC served as the primary source due to its robust citation tracking. To corroborate findings and map the global research landscape, we performed equivalent searches in Scopus and PubMed using the same keyword set, time window, and eligibility criteria. To address potential coverage gaps (e.g., important clinical studies in journals not indexed by WoSCC/Scopus), we additionally screened PubMed for high-quality clinical trials on FD overlapping FGIDs (10). This multi-database strategy enhanced the representativeness and comprehensiveness of the dataset and provided a more realistic picture of the field. However, we acknowledge that publications from the latter half of 2025 may not yet be fully indexed, and counts for 2025 could therefore be underestimated.

### 2.3 Bibliometric analysis

In the bibliometric phase, we integrated metadata on countries/regions, institutions, authors, keywords, and citations. Visualization analyses were conducted using VOSviewer (version 1.6.18), CiteSpace (version 6.2.6), and the bibliometrix R package. Annual and cumulative publication curves were generated in Microsoft Excel 2019 with polynomial fitting. VOSviewer was used to construct and visualize bibliometric networks and overlay maps; in this study it primarily supported analyses of country, institution, and author networks as well as keyword co-occurrence and overlay visualizations. CiteSpace, which employs time-slicing to build temporal networks and integrates them into a panoramic knowledge map, was used for keyword analysis, burst detection, and timeline clustering. Bibliometrix, an open-source R package for scientometrics and bibliometrics, was applied for quantitative analyses implementing major bibliometric techniques.

### 2.4 Cross-database validation

To verify the robustness and completeness of results derived from the WoSCC dataset, additional searches were performed in Scopus and PubMed on the same date (October 1, 2025). The detailed search strategy is presented in Supplementary material 1. Equivalent search strategies-using the same keyword set, Boolean operators, and time window (January 1, 2005 to October 1, 2025)-were adapted to each database’s syntax. The inclusion and exclusion criteria applied to WoSCC were consistently implemented in Scopus and PubMed to ensure comparability (11). For each supplementary database, we extracted the total number of records, annual publication trends, leading contributing countries, and high-frequency author keywords, and compared these with the primary WoSCC dataset. Concordance of temporal trends was assessed using Pearson correlation of annual publication counts across databases. Overlapping and unique records were identified via bibliographic matching based on title, author(s), and publication year. This cross-database comparison was used to confirm whether the thematic foci, geographic distribution, and temporal patterns observed in the WoSCC-based analysis were reproducible across other major bibliographic sources (12). In PubMed, to enhance coverage of clinical research, the query combined Medical Subject Headings (MeSH) with free-text terms to capture additional high-quality clinical studies.

## 3 Results

### 3.1 Publication output and citation trends

**Figure 2:**
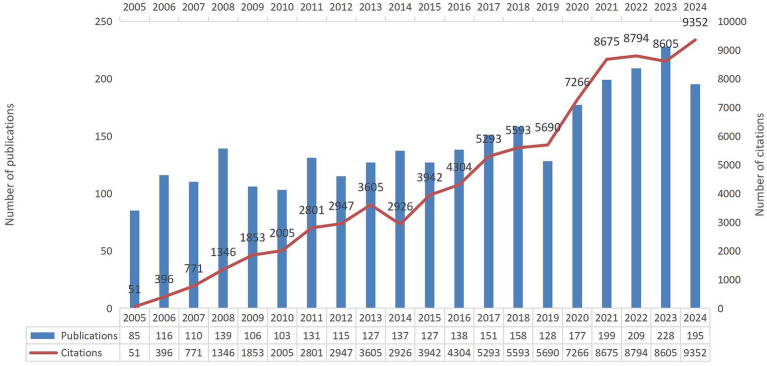
Annual publication output and corresponding citation frequency for studies on functional dyspepsia overlapping other functional gastrointestinal disorders.

Annual publication trends and corresponding citation frequencies are shown in Figure 2. From 2005 to 2024, the number of publications on functional dyspepsia overlapping other functional gastrointestinal disorders exhibited fluctuations but an overall upward trajectory. Note that because data retrieval was completed in September 2025, the full-year output for 2025 was not captured. During this period, both the annual number of publications and the annual citation counts increased rapidly. In terms of citation metrics, the included publications were cited a total of 93,627 times, with an average of 30.9 citations per article. Citations peaked in 2023, when 195 papers were published and accrued 9,352 citations. Overall, publication output continues to grow steadily. These results indicate that research on FD overlapping other FGIDs has received sustained attention over the past two decades, is currently in a rapid growth phase, and is likely to maintain momentum.

### 3.2 Countries/regions

**Figure 3:**
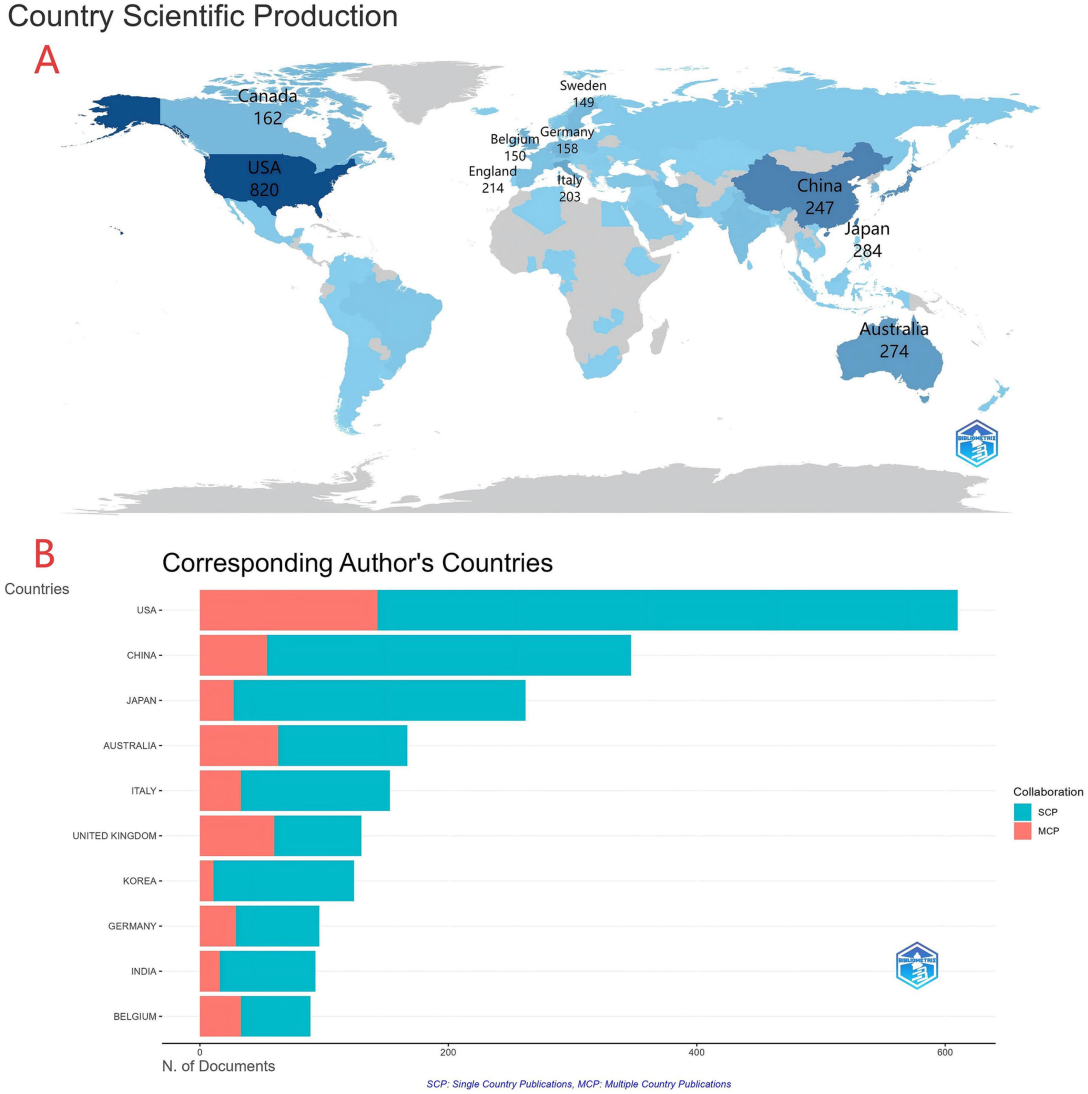
**(A)** Global map of national/regional research output. **(B)** Bar chart of output by corresponding author’s country/region, colored by SCP (single-country publications, cyan) and MCP (multi-country publications, red).

Ninety-eight countries/regions contributed to this field. As summarized in Table 1, distinctive global patterns of research output and collaboration are evident. The United States ranked first with 820 papers (27.06% of all publications), the highest total citations (40,017), and an H-index of 89, underscoring its comprehensive leadership. China ranked second with 347 papers (11.45%), reflecting substantial research scale; however, its citations per paper (38.81) and H-index (46) were relatively lower, suggesting room to improve average impact and top-tier influence. Notably, several medium-sized high-income countries demonstrated excellent efficiency and recognition, including Belgium (69.36 citations per paper), Canada (68.38), England (64.19), and Australia (62.04), each exceeding 60 citations per paper. Australia and England were exemplary in balancing quality and quantity (4th and 5th in output, with H-indexes of 61 and 59, respectively). Traditional research powerhouses such as Japan, Germany, Italy, and Sweden also remained in the top ten with balanced metrics. Overall, the field presents a three-tier pattern: the United States as a dominant hub; China rapidly catching up; and multiple European and Australasian countries distinguished by high-quality outputs. This highlights the need to evaluate research performance across multiple dimensions, including productivity, cumulative impact, and per-article quality. Figures 3A,B display the geographic distribution and bar chart of publications by corresponding author’s country, distinguishing single-country publications (SCP; cyan) from multiple-country publications (MCP; red) to indicate research scale and international collaboration. The United States leads in both total output and SCP (820), reflecting strong domestic productivity and active international collaboration. Countries such as China show substantial domestic output with comparatively lower MCP proportions (347).

**Table 1:** Top 10 productive countries.

Rank	Country	Quantity	Proportion (%)	SOTC	ACI	H-index
1	USA	820	27.06%	40,017	48.80	89
2	China	347	11.45%	10,690	38.81	46
3	Japan	284	9.37%	7,888	27.77	42
4	Australia	274	9.04%	16,998	62.04	61
5	England	214	7.06%	13,736	64.19	59
6	Italy	203	6.70%	11,050	54.43	42
7	Canada	162	5.35%	11,077	68.38	50
8	Germany	158	5.21%	6,068	38.41	41
9	Belgium	150	4.95%	10,404	69.36	48
10	Sweden	149	4.92%	7,230	48.52	40

### 3.3 Authors

From the 3,030 retrieved articles, a total of 13,047 authors were identified. Analysis of the co-authorship network reveals the core author groups in research on FD overlapping other FGIDs. Based on publication counts (Table 2), Professor Nicholas J. Talley (University of Newcastle, Australia) ranks first by a wide margin with 188 papers. His leading H-index (61) and high accumulated citation impact (ACI) further establish his outstanding contributions and scholarly leadership in this field. Jan Tack (KU Leuven, Belgium) and Paul Moayyedi (McMaster University, Canada) exemplify high-quality, highly influential research, with ACI values among the top performers. Geographically, Australia constitutes a notable research cluster (40% of the top 10 authors), particularly centered on the University of Newcastle, indicating strong internal collaboration. Europe (Belgium, the United Kingdom) and North America (the United States, Canada) are also major contributors. In addition, the list includes scholars specializing in the brain-gut axis and biostatistics, suggesting that research foci span epidemiology, pathophysiology, clinical trials, and data modeling.

**Table 2:** Top 10 productive authors.

Rank	Author	Country	Institution	Quantity	ACI	H-index
1	Talley, Nicholas J	Australia	University of Newcastle	188	78.03	61
2	Tack, Jan	Belgium	KU Leuven	95	92.95	41
3	Ford, Alexander	United Kingdom	University of Leeds	48	88.25	28
4	Jones, Michael P	Australia	Macquarie University	47	50.32	25
5	Walker, Marjorie Mary	Australia	University of Newcastle	46	55.37	25
6	Zinsmeister, Alan R	United States	Mayo Clinic	38	75.26	26
7	Moayyedi, Paul	Canada	McMaster University	35	88.83	23
8	Koloski, Natasha	Australia	University of Queensland	33	49.55	19
9	Van Oudenhove, Lukas	Belgium	KU Leuven	32	67.09	23
10	Miwa, Hiroto	Japan	Hyogo Medical University	32	35.13	20

**Figure 4:**
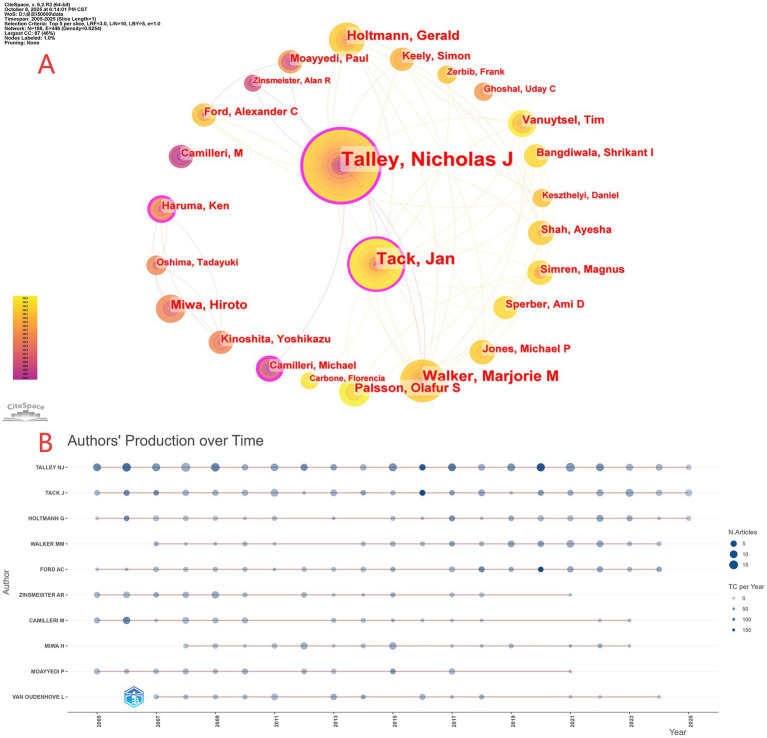
**(A)** Core author collaboration map. **(B)** Temporal visualization of research output and impact.

As shown in Figure 4A, the field is chiefly led by Nicholas J. Talley and Jan Tack, with several characteristic subgroups forming around them: Talley’s team focusing on neurogastroenterology; Tack’s team emphasizing gastrointestinal motility disorders; and an East Asian cluster represented by Miwa and Haruma that prioritizes studies in Asian populations. The overall network density is 0.0254, indicating broad collaboration alongside pronounced cluster differentiation, which corroborates the patterns observed in Figures 4A,B. Figure 4B presents a time-based visualization (2005–2025) with author lists, where dot size encodes the number of publications (three bins: 5, 10, 15) and color intensity reflects annual total citations (four levels: 0–150). This view captures both the consistently high productivity and impact of leading authors (as in Figure 4A) and the rising recent activity of others (e.g., Alexander Ford), thereby elucidating how scholarly output and influence have evolved over time.

### 3.4 Institutions

**Figure 5:**
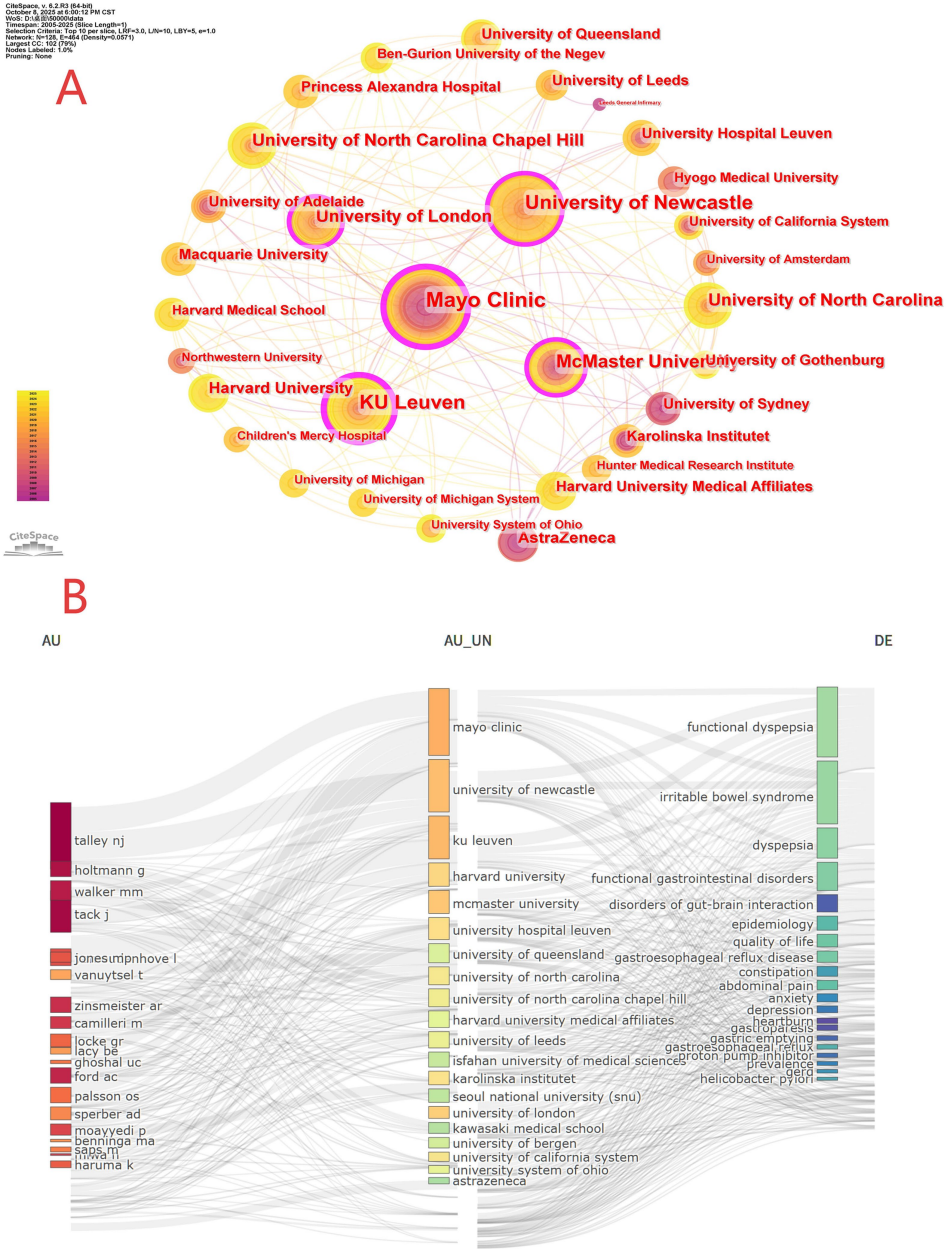
**(A)** Core institutional collaboration network (nodes = institutions; links = collaborations; network density = 0.0571). **(B)** Three-field plot linking core authors, institutions, and keywords.

From the 3,030 retrieved articles, a total of 3,673 institutions were identified. As shown in Table 3, among the top 10 high-output institutions worldwide in research on functional dyspepsia overlapping other functional gastrointestinal disorders, the Mayo Clinic (United States) ranks first with 170 publications and 11,842 total citations, demonstrating outstanding performance in both productivity and scholarly impact (H-index = 57). KU Leuven (Belgium) and the University of Newcastle (Australia) rank second and third, respectively. In terms of research quality, McMaster University (Canada) and the University of Leeds (England) both achieved ACI greater than 77, indicating strong per-article influence, while University Hospital Leuven leads with an ACI of 81.09, underscoring the pronounced visibility of its outputs. Overall, U. S. institutions hold a numerical advantage (three in the top 10), while European institutions perform equally strongly, jointly driving high-level research in this field. Based on CiteSpace, Figure 5A presents a global institutional collaboration network with 128 nodes and 464 links (network density = 0.0571), indicating a highly concentrated structure. Approximately 79% of nodes (102) belong to a dominant cluster, within which the Mayo Clinic acts as the central hub; its larger node size and multi-ring structure highlight sustained influence over the past two decades. The color-time overlay further shows that early activity (2005–2010; deeper purple hues) was led by North American institutions such as the Mayo Clinic and Harvard University; in the mid-period (2011–2018; intermediate tones), European and Canadian institutions (e.g., KU Leuven, McMaster University) progressively integrated and formed regional clusters; and in recent years (2019–2023; brighter yellow), active collaborations have emerged involving industry (e.g., AstraZeneca) and universities (e.g., University of Gothenburg), reflecting a strengthening trend toward industry-academia-research synergy. Overall, the network has evolved from being driven by a few core institutions to a complex, highly integrated innovation system linking multiple regions (North America-Europe, Oceania-Nordic countries). Figure 5B uses a three-field plot with streamlines to depict the close relationships among core authors, research institutions, and keywords. It highlights deep collaborations between key scholars (e.g., Talley NJ, Holtmann G) and leading institutions (e.g., Mayo Clinic, University of Newcastle) across hotspots such as functional dyspepsia overlapping other FGIDs, DGBI, and GERD. It also underscores the field’s expansion toward a multidimensional biopsychosocial model, encompassing intersecting themes such as psychological factors and quality of life.

**Table 3:** Top 10 productive institutions.

Rank	Institution	Country	Quantity	SOTC	ACI	H-index
1	Mayo Clinic	USA	170	11,842	69.66	57
2	KU Leuven	Belgium	123	8,316	67.61	45
3	University of Newcastle	Australia	120	6,944	57.87	40
4	McMaster University	Canada	88	6,835	77.67	37
5	University of London	England	70	3,654	52.20	31
6	University of North Carolina	USA	68	4,144	60.94	28
7	University Hospital Leuven	Belgium	64	5,190	81.09	32
8	Harvard University	USA	56	2,136	38.14	20
9	Karolinska Institutet	Sweden	53	3,054	57.62	26
10	University of Leeds	England	52	4,045	77.79	28

### 3.5 Journals

**Figure 6:**
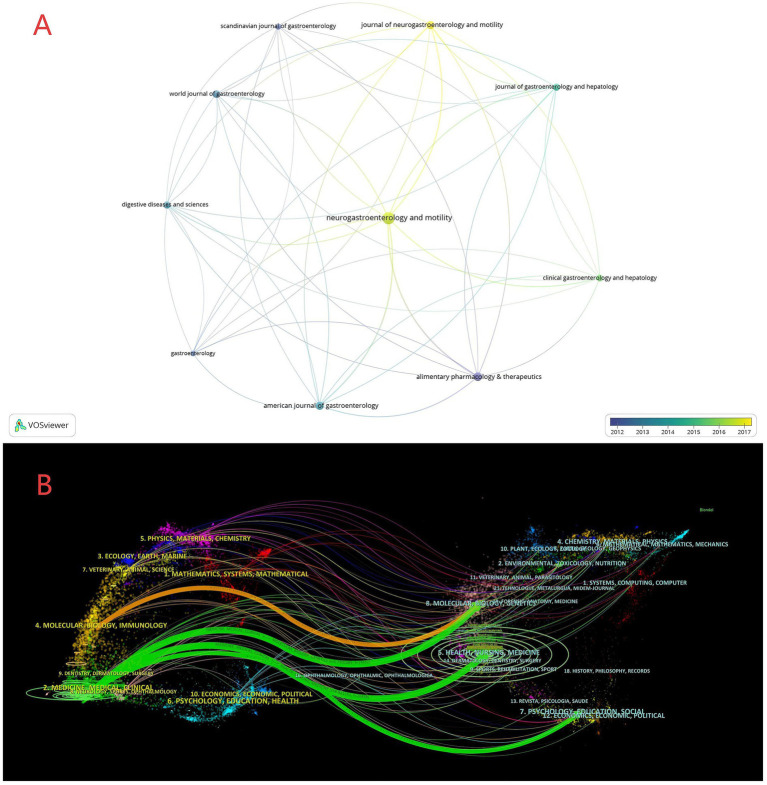
**(A)** Overlay co-citation network of core journals (time gradient). **(B)** Dual-map overlay of citing and cited journals (2005–2025).

As summarized in Table 2, the top 20 journals collectively form a functionally complementary publication ecosystem for research on functional dyspepsia overlapping other functional gastrointestinal disorders. Neurogastroenterology and Motility serves as the core specialty platform with 209 publications, reflecting strong focus on this niche area. Although Gut (IF = 25.8) and Gastroenterology (IF = 25.1) contributed fewer papers (36 and 43, respectively), their average citations per item (ACI = 191.67 and 224.84) place them at the pinnacle of scholarly influence. High-impact Q1 journals such as Alimentary Pharmacology & Therapeutics (106 papers, IF = 6.7) and American Journal of Gastroenterology (96 papers, IF = 8.5) constitute the field’s backbone. The inclusion of Journal of Pediatric Gastroenterology and Nutrition highlights pediatric directions, while the presence of Nutrients (IF = 5.0) signals nutrition as an important cross-disciplinary dimension. Together, these outlets depict a continuum from deep disciplinary specialization to interdisciplinary integration. Figure 6A visualize the journal landscape from one complementary perspective: “associative structure” and “influence density.” In Figure 6A (overlay network), nodes denote core journals in the field (e.g., Scandinavian Journal of Gastroenterology, Journal of Neurogastroenterology and Motility, American Journal of Gastroenterology). Links represent co-citation or topical relatedness, with denser connections indicating more frequent scholarly interaction (for instance, numerous cross-links between Journal of Neurogastroenterology and Motility and Clinical Gastroenterology and Hepatology due to overlapping themes). A yellow-gradient timeline illustrates temporal dynamics of journal activity: for example, World Journal of Gastroenterology appears bluer, reflecting higher activity around 2013, whereas Journal of Gastroenterology and Hepatology trends yellow, indicating heightened activity in 2016–2017. We applied a journal dual-map overlay (Figure 6B) to explore disciplinary structure and identify emerging frontiers by visualizing citation paths between citing and cited journals from 2005 to 2025. The left side represents citing journals (knowledge frontiers), and the right side shows cited journals (knowledge bases). Four principal citation trajectories were identified. The main paths extend from “Molecular/Biology/Immunology” and “Medicine/Medical/Clinical” on the citing side to “Molecular Biology/Genetics” and “Health/Nursing/Medicine” on the cited side. This indicates that authors predominantly publish in biomedical and clinical outlets while drawing extensively on foundational research in molecular biology and genetics. Overall, these findings suggest that research on FD overlapping other FGIDs spans multiple dimensions, including psychosocial science, molecular biology, and clinical disciplines.

### 3.6 Keywords

**Figure 7:**
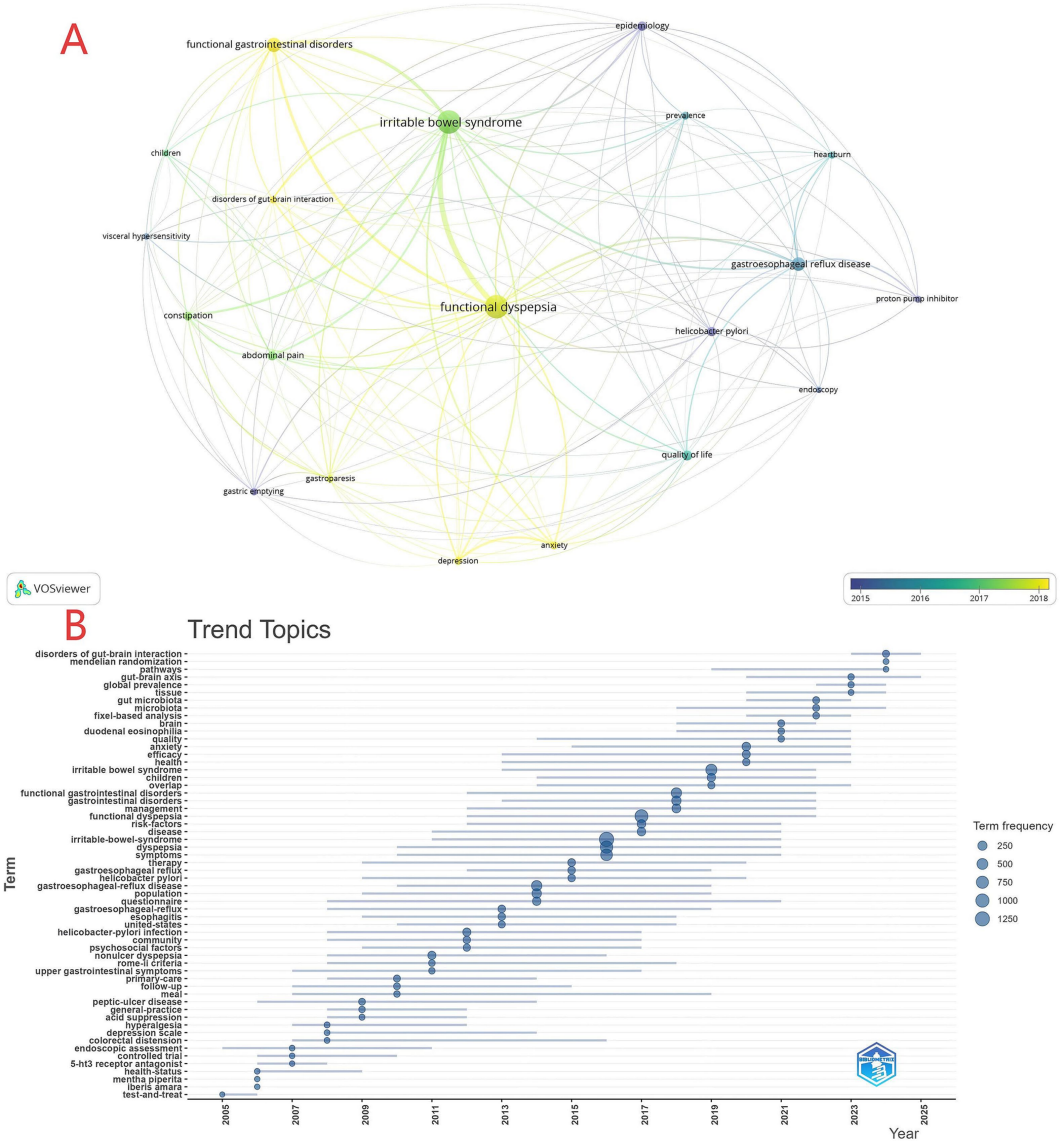
**(A)** Overlay visualization of keyword clusters. **(B)** Timeline of keyword evolution (2005–2025).

Keyword co-occurrence analysis is an effective approach for identifying research hotspots. Based on 5,203 original keywords, we selected 20 high-frequency terms (occurrence ≥ 35) for in-depth analysis. As shown in Table 4, functional dyspepsia (FD; 557) and irritable bowel syndrome (IBS; 557) are the absolute cores, around which a disease cluster emerges that includes gastroesophageal reflux disease (GERD; 196), disorders of gut-brain interaction (DGBI; 59), and gastroparesis (58). The research landscape also encompasses key symptoms-abdominal pain (94), constipation (85), and diarrhea (35); diagnostic/therapeutic approaches-proton pump inhibitors (PPIs; 61), gastric emptying (60), and endoscopy (45); and risk/comorbidity factors-*Helicobacter pylori* (87), anxiety (70), and depression (64). Collectively, these converge on quality of life (QoL; 102) as a central outcome. Children (45) are also highlighted as a special population. This delineates a multidimensional structure spanning disease definitions, symptom management, clinical evaluation and treatment, and contributory factors. Figure 7A portrays the 2015–2018 research ecology from various angles. The influence density map indicates that IBS, FD, and FGIDs represent the most influential hotspots. The thematic association network further shows dense co-occurrence among these core topics and abdominal pain, DGBI, anxiety, and depression, while GERD clusters strongly with *H. pylori* and PPIs. Temporal coloring suggests that interest in FGIDs and psychiatric comorbidities rose markedly in 2017–2018, indicating intensified focus on gut-brain axis mechanisms and psychological factors during this period. The keyword timeline in Figure 7B traces the evolution of hotspots from 2005 to 2025. Early work (2005–2010) focused on traditional targets and strategies such as 5-HT receptors, endoscopic assessment, and acid suppression. The middle period (2010–2015) shifted toward clinical and etiological exploration of GERD, *H. pylori* infection, FD, and IBS. Recent years (2015–2023 and beyond) advance into frontiers including DGBI, the gut microbiota, and the brain-gut axis, while integrating interdisciplinary methods such as Mendelian randomization and global prevalence analyses-signaling concurrent deepening at both the micro-mechanistic and macro-epidemiologic levels.

**Table 4:** Top 20 Keywords.

Rank	Keywords	Counts
1	Irritable Bowel Syndrome	557
2	Functional Dyspepsia	557
3	Functional Gastrointestinal Disorders	218
4	Gastroesophageal Reflux Diseases	196
5	Quality of Life	102
6	Abdominal Pain	94
7	Epidemiology	92
8	*Helicobacter pylori*	87
9	Constipation	85
10	Anxiety	70
11	Prevalence	66
12	Depression	64
13	Proton Pump Inhibitor	61
14	Gastric Emptying	60
15	Disorders of Gut-Brain Interaction	59
16	Gastroparesis	58
17	Heartburn	56
18	Endoscopy	45
19	Children	45
20	Diarrhea	35

**Figure 8:**
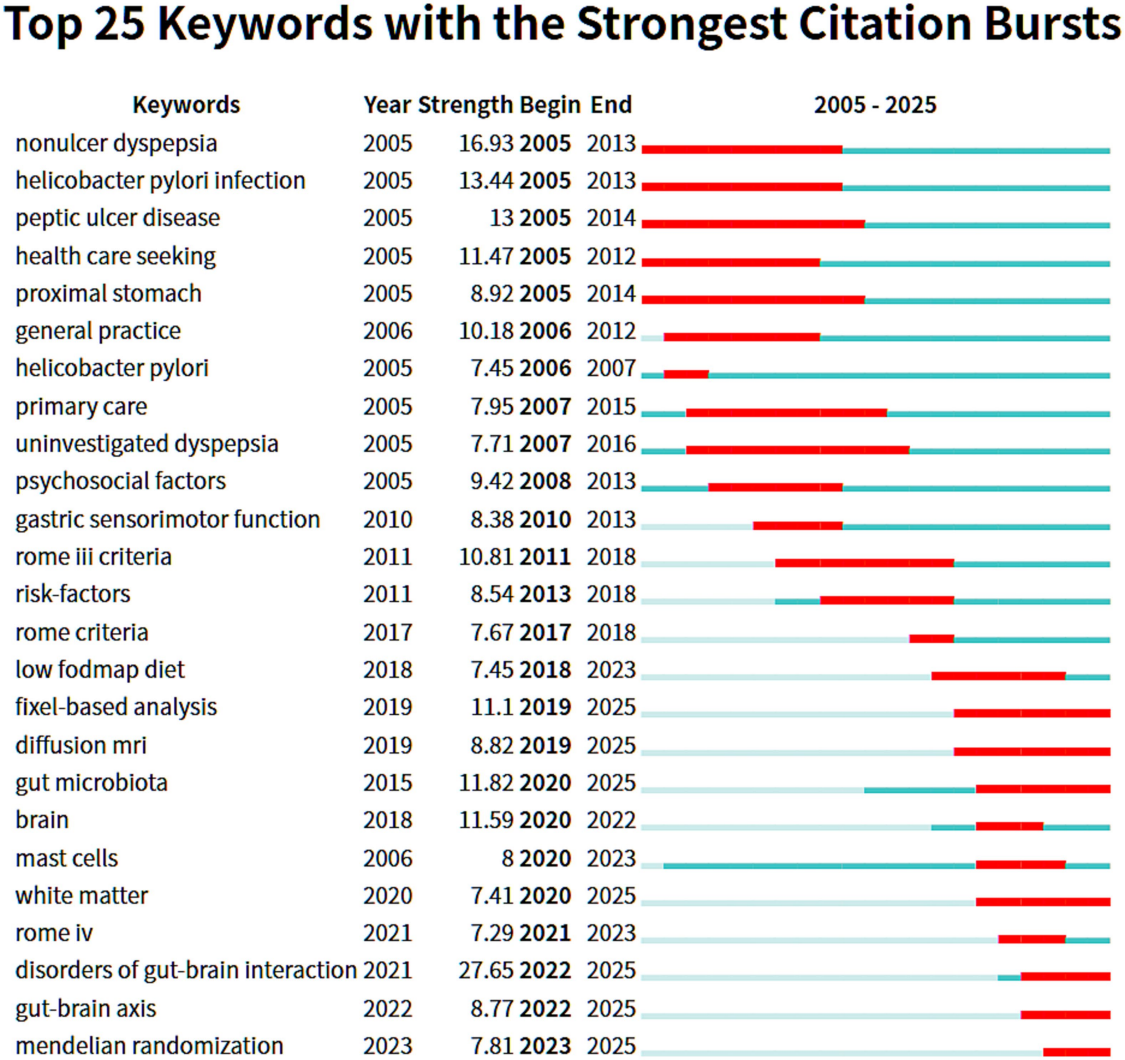
CiteSpace visualization of the top 25 keywords with the strongest citation bursts.

Figure 8's citation burst analysis (top 25 keywords; strength range 7.45–27.65) refines these trends. Early hotspots (2005–2010) centered on non-ulcer dyspepsia and *H. pylori* infection. Subsequent work expanded to primary care pathways, psychosocial factors, and gastric sensorimotor function, with the Rome III criteria (2011–2018) promoting diagnostic standardization. From 2017–2025, frontiers reflect profound cross-disciplinary integration: the low-Fermentable Oligosaccharides, Disaccharides, Monosaccharides and Polyols (FODMAP) diet leads non-pharmacologic interventions; fixel-based analysis and diffusion MRI enable more precise assessment; the gut microbiota, brain-gut axis, and DGBI (burst strength 27.65, the highest) anchor mechanistic inquiry; Mendelian randomization strengthens causal inference; and Rome IV reflects ongoing refinement of disease definitions. Overall, the field has progressed from traditional diagnostics and therapeutics, through mechanistic exploration and clinical standardization, toward deep interdisciplinary integration.

### References

3.7

Table 5's highly cited references delineate the intellectual foundations of “functional dyspepsia overlapping other functional gastrointestinal disorders.” Consensus and breakthroughs converge on disease definitions, shared mechanisms, and integrated management. Foundational definitions and classification: The Rome consensus series (e.g., 41, 50) established unified diagnostic frameworks for FD, IBS, and related FGIDs, formally recognizing the high prevalence of symptom overlap and multimorbidity. Convergent mechanisms: Mechanistic studies elucidate shared pathophysiology across overlapping FGIDs, including bidirectional gut-brain regulation (42), gut microbiota dysbiosis (43), and neurotransmitter dysfunction-particularly serotonergic signaling (44). These findings provide biological explanations for symptom clustering and frequent comorbidity. Population burden and overlap: Epidemiologic work (e.g., 45, 51) quantified global prevalence, overlap patterns (e.g., with GERD and IBS), and clinical burden, reinforcing the need for holistic assessment. Integrated, patient-centered management: Guidance evolved from single-condition care (e.g., *H. pylori* management; 46) to comprehensive, individualized strategies spanning psychosocial comorbidity (47), dietary interventions (48), and complementary approaches (49). Collectively, these high-impact works reflect a shift from siloed disease entities toward a systems view emphasizing mechanistic interconnections, symptom overlap, and integrated care pathways.

**Table 5:** Top 20 cited references.

Rank	Title	Journal	Author	Year	Citation
1	Update on the epidemiology of gastro-esophageal reflux disease: a systematic review	GUT	El-Serag et al. (45)	2014	1,310
2	Functional gastroduodenal disorders	GASTROENTEROLOGY	Tack et al. (41)	2006	1,277
3	Childhood functional gastrointestinal disorders: Child/adolescent	GASTROENTEROLOGY	Rasquin et al. (56)	2006	1,203
4	The serotonin signaling system: From basic understanding to drug development-for functional GI disorders	GASTROENTEROLOGY	Gershon and Tack (44)	2007	1,202
5	ACG Clinical Guideline: Treatment of *Helicobacter pylori* Infection	AMERICAN JOURNAL OF GASTROENTEROLOGY	Chey et al. (46)	2017	1,096
6	Gastroduodenal Disorders	GASTROENTEROLOGY	Stanghellini et al. (50)	2016	988
7	American college of gastroenterology guideline on the management of *Helicobacter pylori* infection	AMERICAN JOURNAL OF GASTROENTEROLOGY	Chey et al. (57)	2007	923
8	Intestinal microbiota in functional bowel disorders: a Rome foundation report	GUT	Simren et al. (43)	2013	680
9	Global prevalence of irritable bowel syndrome according to Rome III or IV criteria: a systematic review and meta-analysis	LANCET GASTROENTEROLOGY & HEPATOLOGY	Oka et al. (51)	2020	529
10	Management of functional somatic syndromes	LANCET	Henningsen et al. (47)	2007	529
11	Development and Validation of the Rome IV Diagnostic Questionnaire for Adults	GASTROENTEROLOGY	Palsson et al. (58)	2016	488
12	Childhood Functional Gastrointestinal Disorders: Child/Adolescent	GASTROENTEROLOGY	Hyams et al. (59)	2016	473
13	Global prevalence of, and risk factors for, gastro-esophageal reflux symptoms: a meta-analysis	GUT	Eusebi et al. (60)	2018	470
14	Ultraprocessed food and chronic noncommunicable diseases: A systematic review and meta-analysis of 43 observational studies	OBESITY REVIEWS	Lane et al. (48)	2021	446
15	The brain-gut pathway in functional gastrointestinal disorders is bidirectional: a 12-year prospective population-based study	GUT	Koloski et al. (42)	2012	440
16	Second Asia-Pacific Consensus Guidelines for *Helicobacter pylori* infection	JOURNAL OF GASTROENTEROLOGY AND HEPATOLOGY	Fock et al. (61)	2009	430
17	ACG and CAG Clinical Guideline: Management of Dyspepsia	AMERICAN JOURNAL OF GASTROENTEROLOGY	Moayyedi et al. (62)	2017	423
18	Presentation and Epidemiology of Gastroesophageal Reflux Disease	GASTROENTEROLOGY	Richter and Rubenstein (63)	2018	403
19	The global prevalence of IBS in adults remains elusive due to the heterogeneity of studies: a Rome Foundation working team literature review	GUT	Sperber et al. (53)	2017	390
20	A review of the bioactivity and potential health benefits of peppermint tea (Mentha piperita L.)	PHYTOTHERAPY RESEARCH	McKay and Blumberg (49)	2006	383

**Figure 9:**
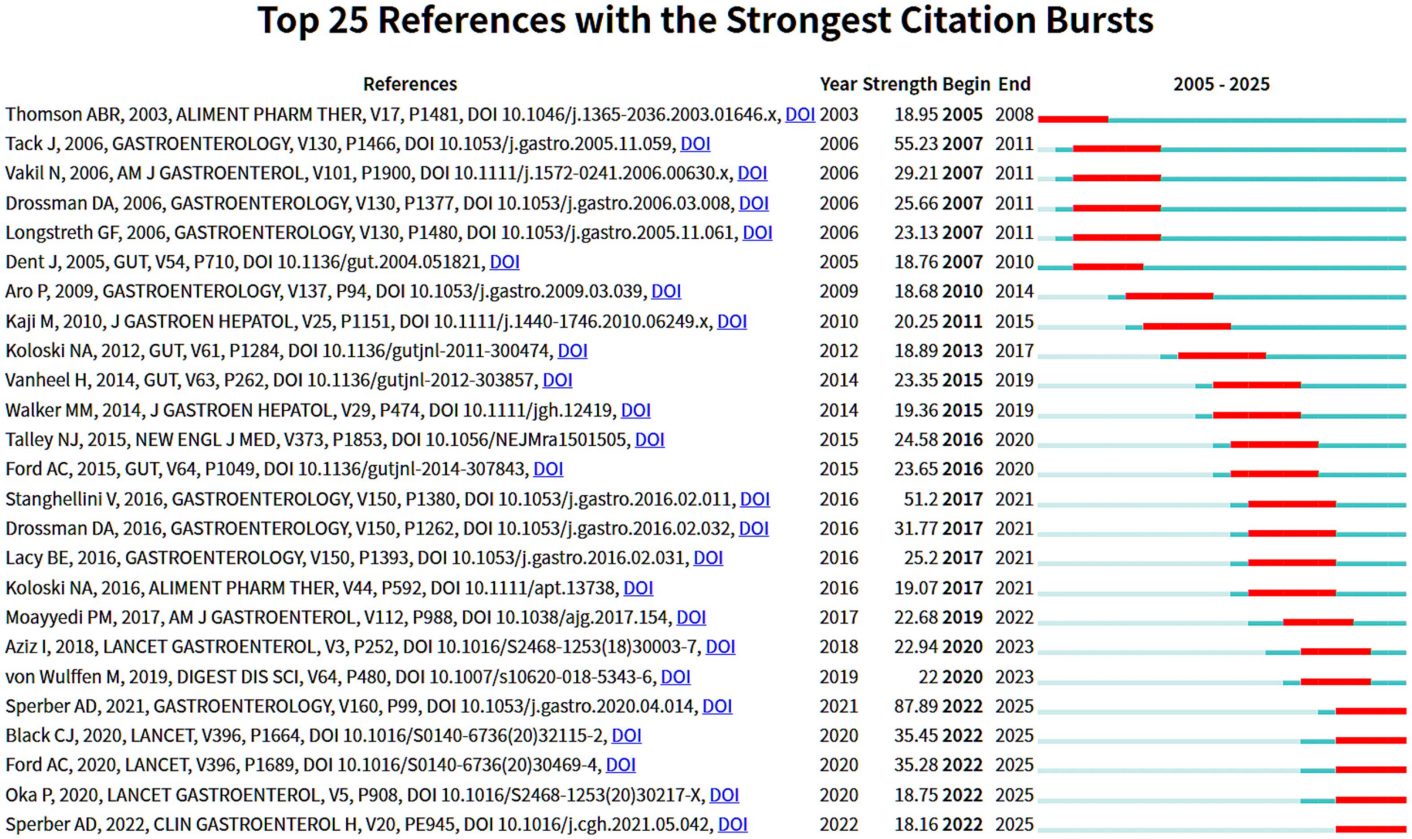
CiteSpace visualization of the top 25 references with the strongest citation bursts (2005–2025).

Figure 9, highlighting the top 25 references with the strongest citation bursts from 2005 to 2025, charts how the evidence base matured: Early foundational phase (2003–2010): Centered on the Rome criteria and diagnostic architecture, with Tack et al. (41; burst strength 55.23) and Drossman (1) and colleagues laying the conceptual groundwork for classifying FGIDs and recognizing overlap. Mid expansion phase (2010–2015): Translation into practice expanded via studies such as Koloski et al. (42) and Walker (52), extending the lens to population characteristics, primary-care pathways, and pragmatic clinical management. Recent frontier phase (2015–2025): Mechanistic deepening and precision-oriented care advanced in parallel. Sperber AD (53; burst strength 87.89, the highest) and Stanghellini et al. (50) underpinned the Rome IV era and consolidated gut-brain interaction frameworks. High-impact, evidence-based guidance (e.g., Black et al. (54); Ford et al. (55) in the Lancet) continued to shape practice through 2025, signaling maturation in overlapped FGID management. Emphases include the gut-brain-microbiome axis, refined diagnostics, and personalized therapeutics. Overall, the trajectory of high-burst references documents a clear progression: from definitional consensus, through exploration of shared mechanisms and real-world implementation, to integrated, individualized, and mechanistically informed management for overlapping FGIDs.

### 3.8 Cross-database validation of results

To verify the robustness of the WoSCC-based findings, we conducted equivalent searches in Scopus and PubMed. Although absolute publication counts differed due to variations in database coverage, the annual publication trends were highly consistent: the Pearson correlations between WoSCC and Scopus and between WoSCC and PubMed were 0.934 and 0.924, respectively, both statistically significant (*p* < 0.001). This indicates that the temporal pattern of disciplinary development observed in WoSCC is generalizable and reliable across databases. High-frequency keyword themes also showed substantial overlap. For example, research has converged on the overlapping phenomena among FD, IBS, and GERD. This consistency is reflected in shared high-frequency symptoms (abdominal pain, constipation/diarrhea), common pathophysiological mechanisms (gut-brain axis dysregulation, psychiatric comorbidity, motility disturbances), and common therapeutic challenges (e.g., the use of proton pump inhibitors). Consequently, consensus and frontiers in the field emphasize moving beyond traditional disease classifications to comprehensive elucidate the complex overlaps and shared mechanisms between FD and other FGIDs.

The leading contributing countries were likewise stable across databases, with the United States and China consistently ranking first and second in publication volume. Across all three datasets, FD with GERD, EPS with IBS, PDS with IBS, and FD with FC were identified as primary topics, reinforcing the main conclusions drawn from the WoSCC analysis. Minor differences in lower-ranked keywords and country/region lists largely reflect database-specific indexing policies and journal coverage. However, no substantive differences emerged in the field’s overall trajectory, thematic priorities, or geographic distribution. These results confirm that the observed trends, key contributors, and thematic structures are not artifacts of a single database but represent stable, reproducible patterns across major bibliographic platforms.

### 3.9 Analysis of clinical progress

A total of 106 clinical trials were retrieved from the PubMed database (Supplementary material). These studies can be broadly categorized into two primary research themes: (1) pharmacological optimization and dietary intervention strategies targeting overlapping symptoms of FGIDs, specifically the comorbidity of FD, GERD, and IBS; and (2) clinical efficacy assessments of psychotherapies and complementary and alternative medicine (CAM), such as Chinese herbal medicine and acupuncture, based on the “gut-brain axis” mechanism.

## 4 Discussion

Employing bibliometric approaches, this study represents the first effort to comprehensive map and visualize research trends concerning FD overlapping with other FGIDs from 2005 to 2025. Our findings not only delineated the two-decade evolutionary trajectory of this field but also elucidated its underlying knowledge architecture, shifts in research hotspots, and patterns of international collaboration. Within this research domain, FD + GERD, EPS + IBS, PDS + IBS, and FD + FC consistently emerge as significant entities across analyses of high-frequency keywords, co-citation clusters, and citation bursts. From a bibliometric perspective, this phenomenon marks a critical challenge facing the field: the transition from single-disease diagnosis and treatment toward addressing complex clinical overlap phenotypes.

The steady growth in annual publications and citations (Figure 2) indicates that the overlap between FD and other FGIDs—particularly IBS and GERD—has evolved into a domain of high academic interest within gastroenterology. This upward trend in literature reflects not only improved clinical recognition but also profoundly reveals a paradigm shift currently underway in academia: a transition from an isolated disease framework based on anatomical location (e.g., stomach vs. intestine) toward a “pan-gastrointestinal dysfunction” model that elucidates shared pathophysiological pathways across organs. Our analysis identified a distinct “core-periphery” structure within research collaborations. Prolific researchers such as Nicholas J. Talley and Jan Tack (Table 2), alongside leading institutions including the Mayo Clinic (USA), KU Leuven (Belgium), and the University of Newcastle (Australia) (Table 3), constitute the academic core of this field (Figures 4, 5). Analysis based on co-authorship networks reveals that multinational collaboration—especially among North America, Europe, and Australia—has formed a tightly knit network. Concurrently, there has been a significant increase in publication output on relevant topics from East Asia (specifically China and Japan), reflecting, to some extent, enhanced investment in research regarding overlapping symptoms in this region. Nevertheless, strengthening international collaboration and elevating the average impact per paper remain key objectives.

Analysis of keyword and reference bursts (Figures 7–9) provides an objective basis for understanding the theoretical evolution of this field. Early hotspots (2005–2010), such as *H. pylori*, acid suppression, and the Rome III classification, have shifted in recent years (2015–present) toward the exploration of shared mechanisms—including the brain-gut axis, gut microbiota, psychiatric comorbidities (e.g., anxiety and depression), and Disorders of Gut-Brain Interaction (DGBI). This bibliometric feature corroborates a shift in research focus from a symptom- and organ-centric morphological model to a more comprehensive “gut-brain-microbiome” biopsychosocial framework. Concurrently, emerging interest in dietary interventions (e.g., the low-FODMAP diet) and the application of Magnetic Resonance (MR) marks a research frontier dedicated to strengthening causal inference through non-pharmacological, multimodal means and exploring the possibility of personalized treatment strategies. Highly cited publications (Table 5) and burst references (Figure 9) collectively constitute the foundational knowledge of the field: the Rome criteria series established the diagnostic foundation, while subsequent research has progressively advanced mechanistic understanding and clinical management. Journal analysis (Table 6; Figure 6) reveals a functionally complementary publishing landscape, comprising specialized core journals (*Neurogastroenterology and Motility*), high-impact general clinical journals (*Gut, Gastroenterology*), and key intermediary journals (*Alimentary Pharmacology & Therapeutics*). The prominence of *Nutrients* further reflects the growing interdisciplinary importance of nutritional science as a potential means of modulating overlapping symptoms within this field. Finally, through cross-validation using Scopus and PubMed, we confirmed that findings derived from WoSCC—including annual publication trends, national contributions, and core keywords—are robust and generalizable. This consistency confirms that the research landscape depicted in this paper is not an artifact of a single database but accurately reflects the authentic global development of the field.

**Table 6:** Top 20 journals.

Rank	Journal	Quantity	ACI	IF (2024)	JCR
1	Neurogastroenterology and Motility	209	27.33	2.90	Q2
2	Alimentary Pharmacology & Therapeutics	106	55.44	6.70	Q1
3	American Journal of Gastroenterology	96	84.54	8.50	Q1
4	Journal of Neurogastroenterology and Motility	89	26.39	3.30	Q2
5	World Journal of Gastroenterology	80	32.11	5.40	Q1
6	Journal of Gastroenterology and Hepatology	72	33.26	3.40	Q2
7	Digestive Diseases and Sciences	71	25.13	2.50	Q2
8	Clinical Gastroenterology and Hepatology	61	54.82	12.00	Q1
9	Scandinavian Journal of Gastroenterology	52	29	1.70	Q3
10	Gastroenterology	43	224.84	25.10	Q1
11	Journal of Clinical Gastroenterology	43	25.88	2.80	Q2
12	BMC Gastroenterology	42	23.45	2.60	Q2
13	Journal of Pediatric Gastroenterology and Nutrition	42	34.12	2.60	Q1
14	European Journal of Gastroenterology & Hepatology	39	16.38	1.80	Q3
15	Journal of Gastroenterology	38	41.37	5.50	Q2
16	Gut	36	191.67	25.80	Q1
17	Digestive Diseases	35	23.97	2.10	Q3
18	Digestion	33	20.36	3.60	Q2
19	PLOS ONE	28	14.61	2.90	Q2
20	Nutrients	27	12.67	5.00	Q1

High-frequency appearances of co-citation clusters reveal that specific overlapping phenotypes (e.g., FD + GERD) have become independent research hotspots. The formation of such clusters is not coincidental but rather reflects a dilemma in clinical taxonomy. For instance, research literature on FD and GERD frequently focuses on overlapping symptoms such as epigastric burning or postprandial fullness, causing the two to be highly entangled in the literature and difficult to separate diagnostically. Bibliometric evidence indicates that discussions revolving around the relationship between pathological esophageal acid reflux (PEAR) and FD symptoms constitute an important knowledge base (13). Rather than merely describing symptom severity, these studies reveal the limitations of current symptom-based classification systems when confronting overlap syndromes: patients with FD-GERD overlap (GERD-D) are consistently characterized in the existing literature as possessing more complex clinical features (e.g., higher rates of anxiety/depression and distinct metabolic profiles), which directly leads to higher healthcare resource utilization (14). Consequently, there has been a surge in studies targeting tools such as the Structured Assessment of Gastrointestinal Symptoms (SAGIS) and the diagnostic value of PPIs trials (15). This bibliometrically reflects the methodological efforts within the academic community to disentangle overlapping phenotypes and overcome the limitations of single-disease diagnostic criteria.

Another notable geographic bibliometric cluster emerges in studies targeting Asian populations, particularly regarding the overlap between IBS and EPS subtype of FD. The emergence of this cluster underscores the heterogeneity in disease presentation between the East and the West, presenting a theoretical challenge to the universality of the Rome classification system, which is derived primarily from Western populations. Epidemiological trends indicate that literature pertaining to Asia not only addresses the rising prevalence of IBS but also places greater emphasis on characterizing clinical features predominated by upper gastrointestinal symptoms (16). Validation studies employing factor analysis have identified specific symptom clusters (e.g., epigastric pain with constipation and meal-related intestinal symptoms), which reinforces the intrinsic connection between EPS and IBS within the literature structure (17). These bibliometric findings carry significant theoretical implications: they indicate that the demarcation between IBS and EPS is frequently blurred within Asian populations. This suggests that future taxonomic endeavors should prioritize the development of culturally adapted symptom assessment tools and diagnostic frameworks tailored specifically for Asian patients, rather than rigidly applying existing binary classifications.

Furthermore, the overlap between PDS and IBS constitutes a core component of the literature exploring “shared pathophysiological mechanisms.” Research in this domain primarily focuses on employing experimental methodologies to quantify overlapping symptoms, thereby attempting to dismantle organ-specific boundaries. For instance, experimental studies utilizing the lactulose hydrogen breath test combined with a liquid meal challenge (18), as well as the standardized nutrient challenge test (19), are repeatedly cited within the literature. This is not merely because they delineate the consistency of symptoms—such as abdominal pain and bloating—between PDS and IBS, but fundamentally because they provide empirical support for the hypothesis that “visceral hypersensitivity and brain-gut axis dysregulation serve as shared trans-diagnostic mechanisms.” Community-based epidemiological surveys and derived factor analyses (20) have further established an evidentiary basis within the literature for the intrinsic link between upper and lower gastrointestinal manifestations. The clustering of these studies suggests that future research designs should shift toward phenotype-based clinical trials rather than single-diagnosis trials, in order to more accurately capture these shared pathophysiological characteristics.

The bibliometric cluster regarding FD and FC overlap represents the academic exploration of a “pan-enteric dysmotility” model. Literature evidence demonstrates a high-frequency co-occurrence between gastroparesis-like symptoms and constipation, as well as delayed colonic transit (21). The bibliometric structure of this field indicates that researchers are attempting to construct a dysmotility spectrum spanning the stomach and colon through correlation analyses (e.g., the correlation between constipation severity and the intensity of gastroparesis-like symptoms). Epidemiological studies in Asian populations (16) and analyses of symptom distribution across IBS subtypes (22) have further enriched this cluster, suggesting that the overlap of FD and FC likely shares pathogenic mechanisms involving gastrointestinal dysmotility and visceral hypersensitivity. This provides a theoretical basis for conceptualizing upper and lower gastrointestinal dysmotility as a continuum.

The explosive growth in emerging methodological literature—encompassing Mendelian randomization (MR), microbiome sequencing, and neuroimaging—reflects the field’s shift toward employing multi-omics approaches to disentangle “shared mechanisms” from “disease-specific mechanisms.” Evidence indicates that MR studies are currently deconstructing the causal associations between FD, IBS, and GERD from a genetic perspective. At the microbiome level, the literature has highlighted specific bacterial genera (e.g., Lachnospiraceae NK4A136 group) as potential shared pathological substrates (23). The growing prominence of endocrine (24) and psychological research (25) within the literature further corroborates the central role of the brain-gut axis in mediating the comorbidity between depression and FGIDs. Notably, bibliometric analysis also captures attempts to differentiate specific phenotypes: for instance, studies on metabolic profiles (26) and neuroimaging (25) have revealed subtle distinctions in cortical thickness or biochemical markers across different FGIDs. Rather than simply conflating these conditions, these findings delineate a complex landscape wherein FD and other FGIDs share core pathological mechanisms (e.g., brain-gut axis dysregulation) while simultaneously retaining disease-specific characteristics. This structural evolution of the literature strongly suggests that future diagnostic frameworks must integrate biomarkers to achieve more precise phenotyping.

Thematic concentration on therapeutic research for overlap syndromes is evident in the literature, reflecting an urgent clinical demand for “trans-diagnostic” therapies. Rather than simply emphasizing the absolute efficacy of a single agent, bibliometric data illustrate how interventions targeting specific mechanisms are being broadly explored across different overlapping phenotypes. For example, a large-scale, population-based study spanning 13 years (27) became highly cited because it quantified the immense disease burden imposed by overlapping gastrointestinal reflux and dyspepsia, thereby establishing the necessity of comprehensive treatment. Against this backdrop, research on secretagogues and sensitizing agents (e.g., linaclotide) (28) has extended to patients with FD overlap, aiming to explore their cross-regional effects on upper gastrointestinal symptoms. Similarly, literature on potassium-competitive acid blockers (e.g., vonoprazan) (29) focuses not only on acid suppression but also on the broad improvement of overlapping functional gastrointestinal symptoms, suggesting the universality of acid-related mechanisms in symptom overlap. Studies on prokinetics (e.g., acotiamide) combined with PPIs (30) constitute a bibliometric cluster targeting the composite “motility-acid” mechanism. Furthermore, the growth in literature regarding behavioral therapies and nutritional interventions—such as biofeedback training to correct dyssynergic defecation for FD symptom improvement, and research on nutritional interventions (e.g., specific probiotics) (31)—further enriches this theme. Review literature on pediatric dietary interventions (e.g., low-FODMAP, Mediterranean diet) (32) reflects the field’s cautious approach in the absence of conclusive evidence. Overall, the aggregation of these therapeutic studies is no longer merely about verifying the efficacy of single drugs; rather, it represents a paradigm shift from “symptomatic treatment” toward targeting shared pathophysiological mechanisms such as the “gut-brain-microbiome” axis and “motility-visceral hypersensitivity.”

A bibliometric review of 106 clinical trials from the PubMed database highlights the evolving landscape of clinical management for FD, GERD and IBS, revealing a distinct trend toward optimizing interventions for symptom overlap and leveraging brain-gut axis mechanisms. Given the prevalence of comorbid symptoms where single-mechanism agents often prove inadequate, research has pivoted toward novel pharmacological and dietary strategies. In the pharmacological domain, the potassium-competitive acid blocker Vonoprazan has demonstrated superior efficacy in improving gastrointestinal symptoms in GERD patients, particularly those with erosive esophagitis and obesity (29), while the acetylcholinesterase inhibitor Acotiamide not only enhances gastric motility in FD but also ameliorates epigastric symptoms in PPI-refractory NERD (33), showing favorable tolerability in overlap cases (30). Complementing these pharmaceutical advances, dietary interventions such as Gold kiwifruit have been confirmed to alleviate constipation (34), and the low-FODMAP diet has shown differential efficacy in pediatric populations, yielding better responses in IBS and FD compared to functional abdominal pain (35). Concurrently, the deepening understanding of the brain-gut axis has validated the efficacy of psychomodulation and Complementary and Alternative Medicine (CAM). For instance, the Japanese Kampo medicine “Rikkunshito” was found to simultaneously improve postprandial fullness and anxiety in FD patients (36), and electroacupuncture demonstrated efficacy comparable to loperamide in diarrhea-predominant IBS (37). Furthermore, in pediatric functional abdominal pain, both Internet-delivered Cognitive Behavioral Therapy (Internet-CBT) (38) and low-dose Amitriptyline (39) have proven effective in reducing symptom severity and improving quality of life, while specific Bifidobacterium mixtures offered significant relief for abdominal pain in children with IBS, distinct from their limited effect in FD (40).

Several limitations of this study must be acknowledged. First, the literature search was restricted to English-language publications, which may have omitted significant non-English contributions and introduced potential language bias. Second, cross-national and cross-institutional comparisons based on citation metrics may be influenced by variations in publication dates (time-accumulation bias). Although Total Citations and Average Citations per Item (ACI) were utilized, our analysis lacks field-normalized metrics or adjustments using fixed citation windows. Third, results from co-occurrence analysis, clustering, and citation burst detection are inherently influenced by algorithmic choices and parameter settings, introducing a degree of semantic and methodological subjectivity. Finally, while bibliometric indicators such as citation counts, H-index, and betweenness centrality provide valuable insights into academic influence and network positioning, these quantitative metrics do not directly capture the intrinsic academic quality, innovation, or translational potential of individual studies. Future research should aim to incorporate multilingual literature sources, adopt standardized evaluation frameworks, and employ more comprehensive impact metrics to further advance the field in a robust and nuanced manner.

## 5 Conclusion

Research on the overlap between FD and other FGIDs has evolved into a cohesive and increasingly mature field. Bibliometric evidence clearly indicates a shift in research focus from the diagnosis and management of individual diseases toward elucidating the underlying mechanisms of multimorbidity, with a distinct trajectory toward topics such as the gut-brain axis, gut microbiota, and psychiatric comorbidities. This evolution has catalyzed several distinct research directions. Internationally, collaboration has formed a multi-center network anchored by North America, Europe, and Australia, while the research influence of Asian countries is steadily growing. This global research network not only reflects the growth in academic output but also highlights challenges posed to existing diagnostic criteria (e.g., Rome criteria) by differences in clinical phenotypes between the East and the West. Future efforts should prioritize the integration of multilingual literature and the development of more robust evaluation frameworks. More importantly, trends in literature evolution suggest that future research designs should transcend the single-organ perspective and shift toward conducting phenotype-based clinical trials targeting shared pathological mechanisms. This will facilitate deciphering the molecular basis of specific overlapping phenotypes and developing comprehensive therapies targeting the gut-brain-microbiome axis, ultimately propelling the field from mere symptom control toward etiology-driven precision management.

## Data Availability

The raw data supporting the conclusions of this article will be made available by the authors, without undue reservation.
