# Supplementary material for: Functional dyspepsia overlapping with other functional gastrointestinal disorders: a bibliometric and visualization analysis
Source: Front Med (Lausanne). 2026 Apr 8;13:1742579. doi: 10.3389/fmed.2026.1742579 (PMC13101434; doi:10.3389/fmed.2026.1742579)
Supplement: Supplementary file 1 [file Table_1.DOCX]

**Search Strategy**

**WosCC**

TS=( ("Dyspepsia*" OR "Indigestion*" OR "functional dyspepsia" OR FD OR "non-ulcer dyspepsia" OR "idiopathic dyspepsia" OR "non-organic dyspepsia" OR "non-ulcerative dyspepsia") AND ("gastroesophageal reflux disease" OR GERD OR "irritable bowel syndrome" OR IBS OR "functional constipation" OR FC) ) AND PY=(2005-2025) AND DT=(Article OR Review) AND LA=(English)

**Pubmed**

(("Dyspepsia*"[Title/Abstract] OR "Indigestion*"[Title/Abstract] OR "functional dyspepsia"[Title/Abstract] OR "FD"[Title/Abstract] OR "non-ulcer dyspepsia"[Title/Abstract] OR "idiopathic dyspepsia"[Title/Abstract] OR "non-organic dyspepsia"[Title/Abstract] OR "non-ulcerative dyspepsia"[Title/Abstract]) AND ("gastroesophageal reflux disease"[Title/Abstract] OR "GERD"[Title/Abstract] OR "irritable bowel syndrome"[Title/Abstract] OR "IBS"[Title/Abstract] OR "functional constipation"[Title/Abstract] OR "FC"[Title/Abstract])) AND 2005:2025[dp] AND English[la] AND (Clinical Trial[ptyp] OR Randomized Controlled Trial[ptyp])

**Scopus**

( TITLE-ABS-KEY ( ( "Dyspepsia*" OR "Indigestion*" OR "functional dyspepsia" OR fd OR "non-ulcer dyspepsia" OR "idiopathic dyspepsia" OR "non-organic dyspepsia" OR "non-ulcerative dyspepsia" ) ) AND TITLE-ABS-KEY ( ( "gastroesophageal reflux disease" OR gerd OR "irritable bowel syndrome" OR ibs OR "functional constipation" OR fc ) ) ) AND PUBYEAR > 2004 AND PUBYEAR < 2026 AND ( LIMIT-TO ( DOCTYPE , "ar" ) OR LIMIT-TO ( DOCTYPE , "re" ) ) AND ( LIMIT-TO ( LANGUAGE , "English" ) )
